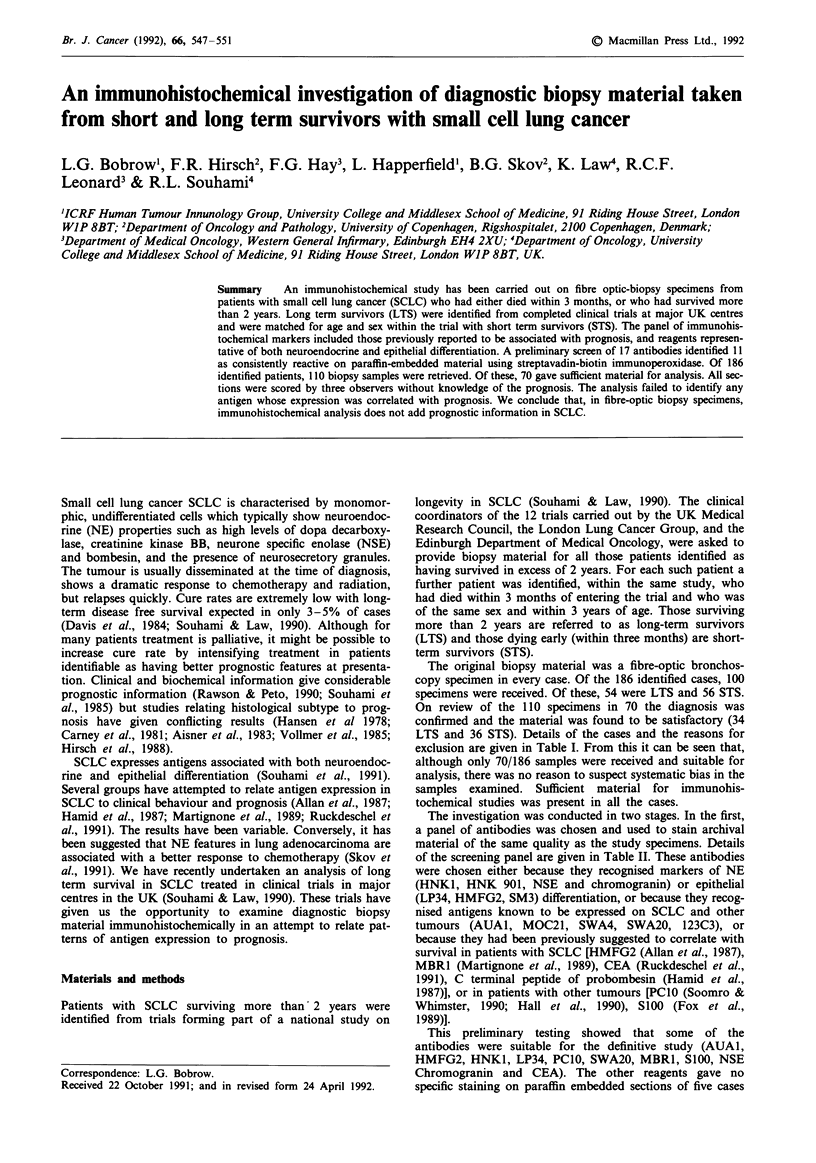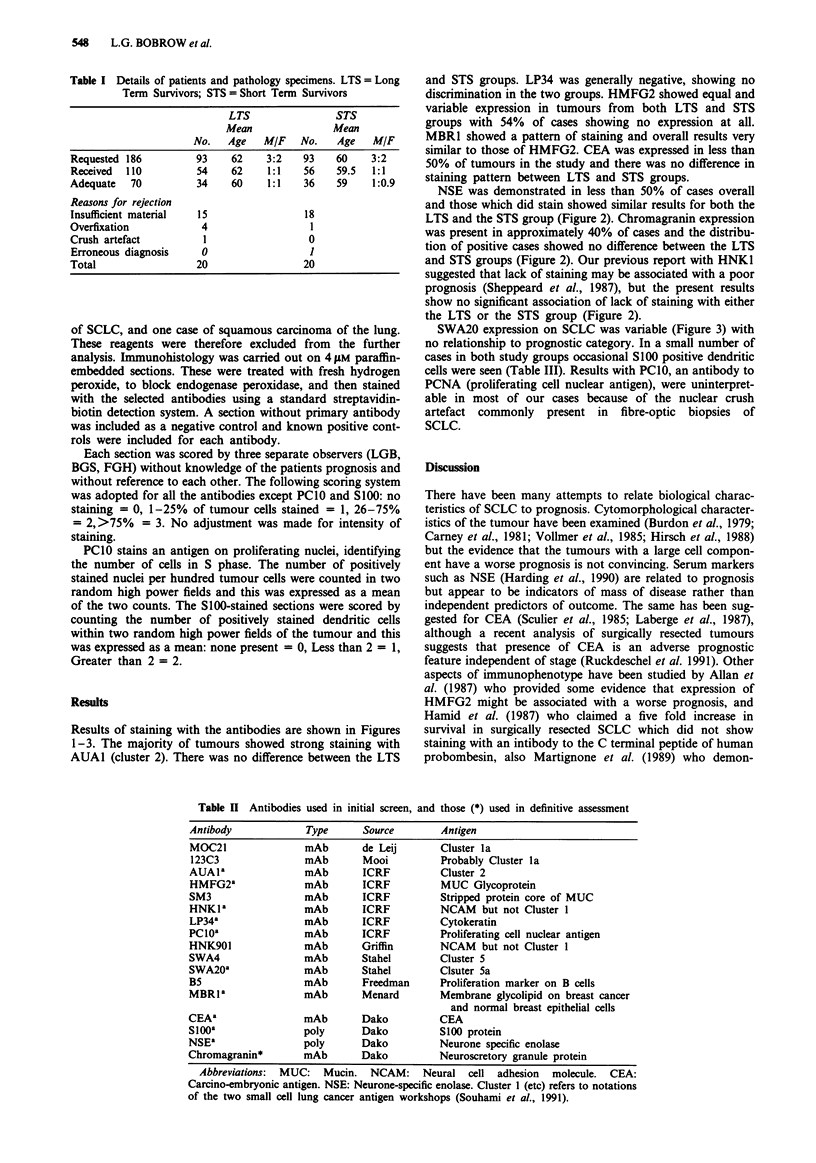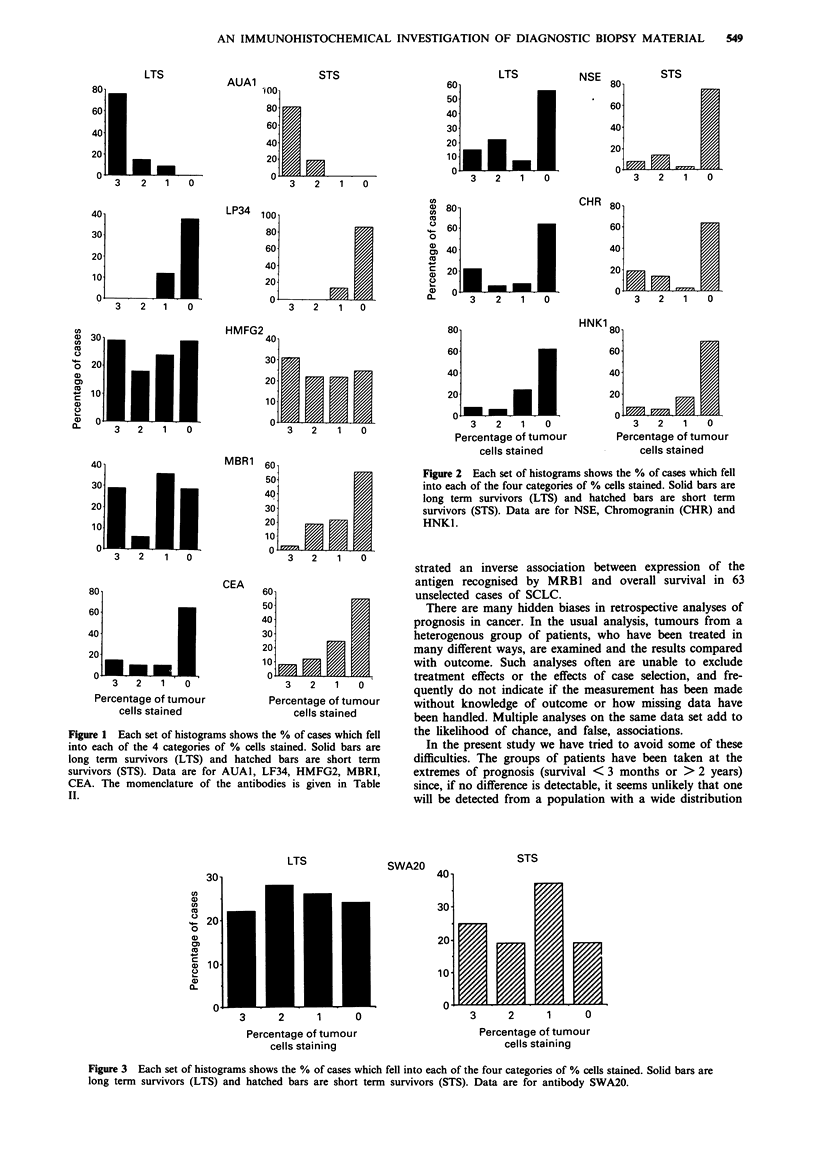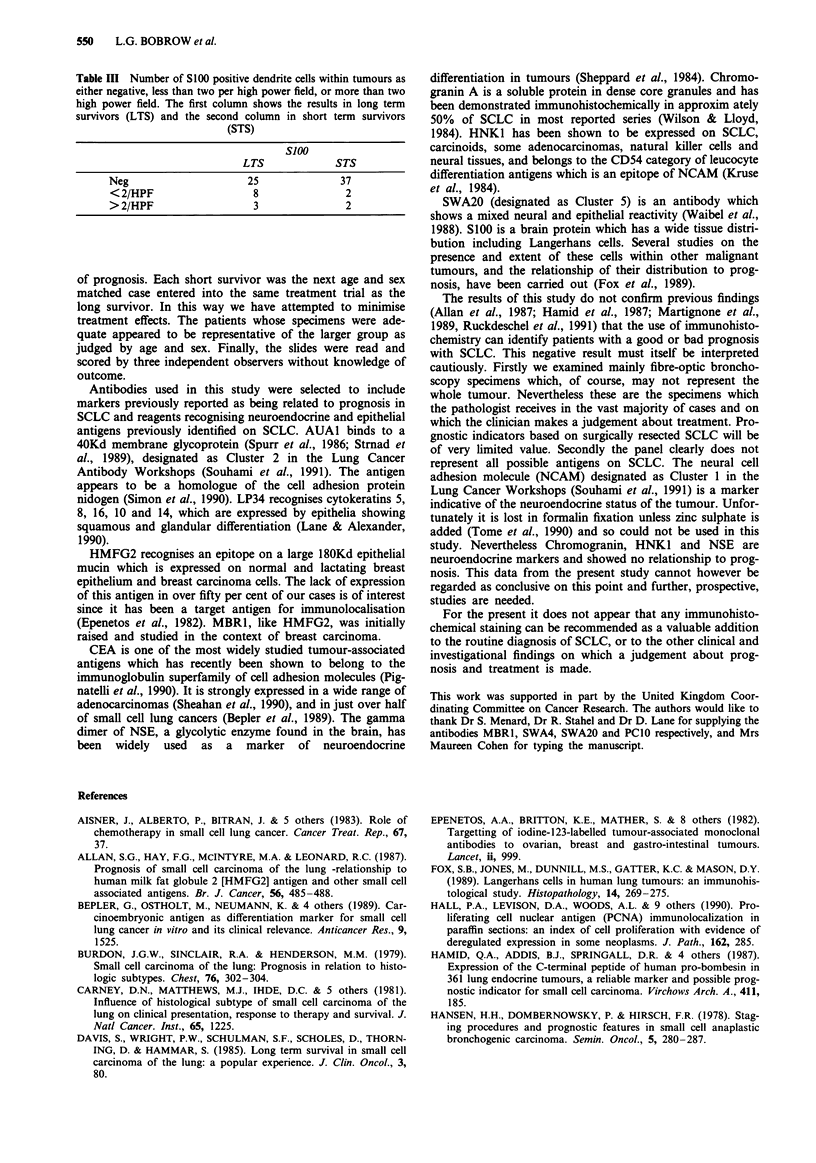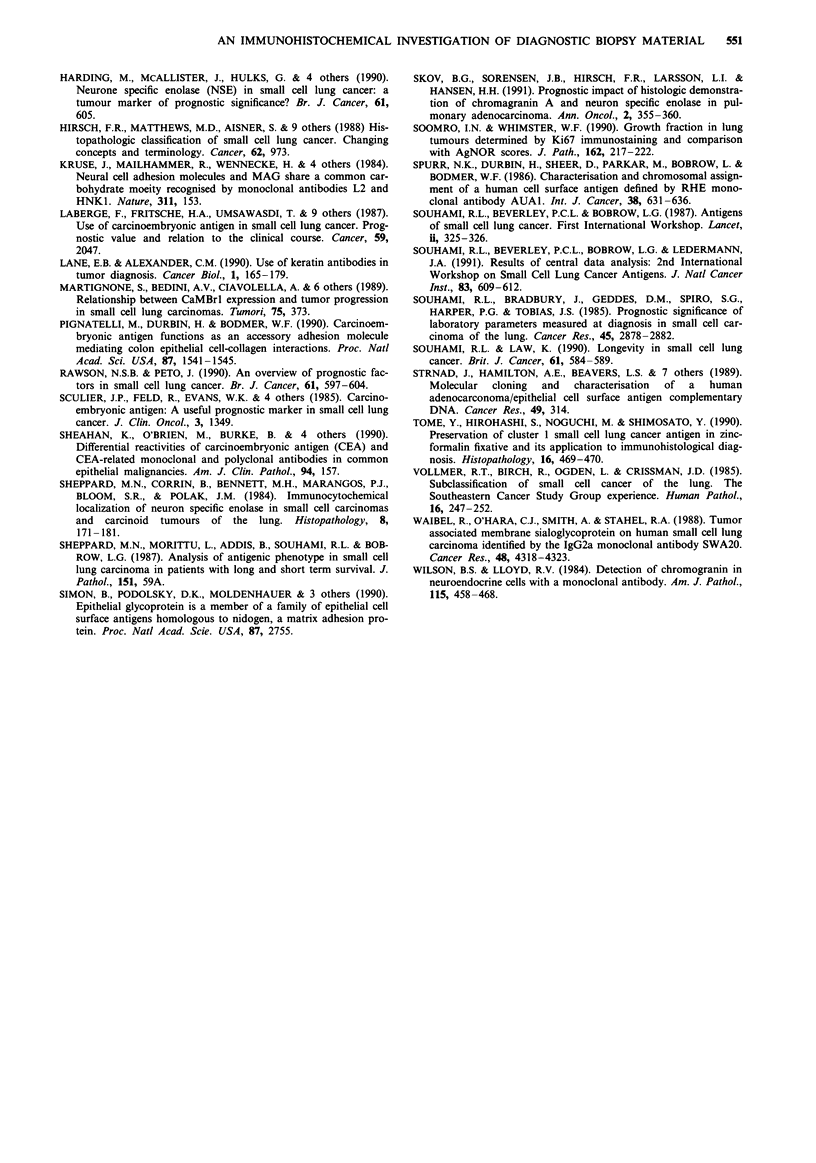# An immunohistochemical investigation of diagnostic biopsy material taken from short and long term survivors with small cell lung cancer.

**DOI:** 10.1038/bjc.1992.311

**Published:** 1992-09

**Authors:** L. G. Bobrow, F. R. Hirsch, F. G. Hay, L. Happerfield, B. G. Skov, K. Law, R. C. Leonard, R. L. Souhami

**Affiliations:** ICRF Human Tumour Immunology Group, University College and Middlesex School of Medicine, London, UK.

## Abstract

An immunohistochemical study has been carried out on fibre optic-biopsy specimens from patients with small cell lung cancer (SCLC) who had either died within 3 months, or who had survived more than 2 years. Long term survivors (LTS) were identified from completed clinical trials at major UK centres and were matched for age and sex within the trial with short term survivors (STS). The panel of immunohistochemical markers included those previously reported to be associated with prognosis, and reagents representative of both neuroendocrine and epithelial differentiation. A preliminary screen of 17 antibodies identified 11 as consistently reactive on paraffin-embedded material using streptavadin-biotin immunoperoxidase. Of 186 identified patients, 110 biopsy samples were retrieved. Of these, 70 gave sufficient material for analysis. All sections were scored by three observers without knowledge of the prognosis. The analysis failed to identify any antigen whose expression was correlated with prognosis. We conclude that, in fibre-optic biopsy specimens, immunohistochemical analysis does not add prognostic information in SCLC.


Br. J. Cancer (1992), 66, 547–551 © Macmillan Press Ltd., 1992

### An immunohistochemical investigation of diagnostic biopsy material taken from short and long term survivors with small cell lung cancer

L.G. Bobrow[1], F.R. Hirsch[2], F.G. Hay[3], L. Happerfield[1], B.G. Skov[2], K. Law[4], R.C.F. Leonard[3] & R.L. Souhami[4]

[1]ICRF Human Tumour Innunology Group, University College and Middlesex School of Medicine, 91 Riding House Street, London W1P 8BT; [2]Department of Oncology and Pathology, University of Copenhagen, Rigshospitalet, 2100 Copenhagen, Denmark; [3]Department of Medical Oncology, Western General Infirmary, Edinburgh EH4 2XU; [4]Department of Oncology, University College and Middlesex School of Medicine, 91 Riding House Street, London W1P 8BT, UK.

**Summary** An immunohistochemical study has been carried out on fibre optic-biopsy specimens from patients with small cell lung cancer (SCLC) who had either died within 3 months, or who had survived more than 2 years. Long term survivors (LTS) were identified from completed clinical trials at major UK centres and were matched for age and sex within the trial with short term survivors (STS). The panel of immunohistochemical markers included those previously reported to be associated with prognosis, and reagents representative of both neuroendocrine and epithelial differentiation. A preliminary screen of 17 antibodies identified 11 as consistently reactive on paraffin-embedded material using streptavadin-biotin immunoperoxidase. Of 186 identified patients, 110 biopsy samples were retrieved. Of these, 70 gave sufficient material for analysis. All sections were scored by three observers without knowledge of the prognosis. The analysis failed to identify any antigen whose expression was correlated with prognosis. We conclude that, in fibre-optic biopsy specimens, immunohistochemical analysis does not add prognostic information in SCLC.

Small cell lung cancer SCLC is characterised by monomorphic, undifferentiated cells which typically show neuroendocrine (NE) properties such as high levels of dopa decarboxylase, creatinine kinase BB, neurone specific enolase (NSE) and bombesin, and the presence of neurosecretory granules. The tumour is usually disseminated at the time of diagnosis, shows a dramatic response to chemotherapy and radiation, but relapses quickly. Cure rates are extremely low with long-term disease free survival expected in only 3–5% of cases (Davis *et al.*, 1984; Souhami & Law, 1990). Although for many patients treatment is palliative, it might be possible to increase cure rate by intensifying treatment in patients identifiable as having better prognostic features at presentation. Clinical and biochemical information give considerable prognostic information (Rawson & Peto, 1990; Souhami *et al.*, 1985) but studies relating histological subtype to prognosis have given conflicting results (Hansen *et al* 1978; Carney *et al.*, 1981; Aisner *et al.*, 1983; Vollmer *et al.*, 1985; Hirsch *et al.*, 1988).

SCLC expresses antigens associated with both neuroendocrine and epithelial differentiation (Souhami *et al.*, 1991). Several groups have attempted to relate antigen expression in SCLC to clinical behaviour and prognosis (Allan *et al.*, 1987; Hamid *et al.*, 1987; Martignone *et al.*, 1989; Ruckdeschel *et al.*, 1991). The results have been variable. Conversely, it has been suggested that NE features in lung adenocarcinoma are associated with a better response to chemotherapy (Skov *et al.*, 1991). We have recently undertaken an analysis of long term survival in SCLC treated in clinical trials in major centres in the UK (Souhami & Law, 1990). These trials have given us the opportunity to examine diagnostic biopsy material immunohistochemically in an attempt to relate patterns of antigen expression to prognosis.

### Materials and methods

Patients with SCLC surviving more than 2 years were identified from trials forming part of a national study on longevity in SCLC (Souhami & Law, 1990). The clinical coordinators of the 12 trials carried out by the UK Medical Research Council, the London Lung Cancer Group, and the Edinburgh Department of Medical Oncology, were asked to provide biopsy material for all those patients identified as having survived in excess of 2 years. For each such patient a further patient was identified, within the same study, who had died within 3 months of entering the trial and who was of the same sex and within 3 years of age. Those surviving more than 2 years are referred to as long-term survivors (LTS) and those dying early (within three months) are short-term survivors (STS).

The original biopsy material was a fibre-optic bronchoscopy specimen in every case. Of the 186 identified cases, 100 specimens were received. Of these, 54 were LTS and 56 STS. On review of the 110 specimens in 70 the diagnosis was confirmed and the material was found to be satisfactory (34 LTS and 36 STS). Details of the cases and the reasons for exclusion are given in Table I. From this it can be seen that, although only 70/186 samples were received and suitable for analysis, there was no reason to suspect systematic bias in the samples examined. Sufficient material for immunohistochemical studies was present in all the cases.

The investigation was conducted in two stages. In the first, a panel of antibodies was chosen and used to stain archival material of the same quality as the study specimens. Details of the screening panel are given in Table II. These antibodies were chosen either because they recognised markers of NE (HNK1, HNK 901, NSE and chromogranin) or epithelial (LP34, HMFG2, SM3) differentiation, or because they recognised antigens known to be expressed on SCLC and other tumours (AUA1, MOC21, SWA4, SWA20, 123C3), or because they had been previously suggested to correlate with survival in patients with SCLC [HMFG2 (Allan *et al.*, 1987), MBR1 (Martignone *et al.*, 1989), CEA (Ruckdeschel *et al.*, 1991), C terminal peptide of probombesin (Hamid *et al.*, 1987)], or in patients with other tumours [PC10 (Soomro & Whimster, 1990; Hall *et al.*, 1990), S100 (Fox *et al.*, 1989)].

This preliminary testing showed that some of the antibodies were suitable for the definitive study (AUA1, HMFG2, HNK1, LP34, PC10, SWA20, MBR1, S100, NSE Chromogranin and CEA). The other reagents gave no specific staining on paraffin embedded sections of five cases

Correspondence: L.G. Bobrow.
Received 22 October 1991; and in revised form 24 April 1992.

**Table I** Details of patients and pathology specimens. LTS = Long Term Survivors; STS = Short Term Survivors

| | *LTS No.* | *LTS Mean Age* | *LTS M/F* | *STS No.* | *STS Mean Age* | *STS M/F* |
|---|---|---|---|---|---|---|
| Requested 186 | 93 | 62 | 3:2 | 93 | 60 | 3:2 |
| Received 110 | 54 | 62 | 1:1 | 56 | 59.5 | 1:1 |
| Adequate 70 | 34 | 60 | 1:1 | 36 | 59 | 1:0.9 |
| *Reasons for rejection* | | | | | | |
| Insufficient material | 15 | | | 18 | | |
| Overfixation | 4 | | | 1 | | |
| Crush artefact | 1 | | | 0 | | |
| Erroneous diagnosis | *0* | | | *1* | | |
| Total | 20 | | | 20 | | |

of SCLC, and one case of squamous carcinoma of the lung. These reagents were therefore excluded from the further analysis. Immunohistology was carried out on 4 μM paraffin-embedded sections. These were treated with fresh hydrogen peroxide, to block endogenase peroxidase, and then stained with the selected antibodies using a standard streptavidin-biotin detection system. A section without primary antibody was included as a negative control and known positive controls were included for each antibody.

Each section was scored by three separate observers (LGB, BGS, FGH) without knowledge of the patients prognosis and without reference to each other. The following scoring system was adopted for all the antibodies except PC10 and S100: no staining = 0, 1–25% of tumour cells stained = 1, 26–75% = 2, >75% = 3. No adjustment was made for intensity of staining.

PC10 stains an antigen on proliferating nuclei, identifying the number of cells in S phase. The number of positively stained nuclei per hundred tumour cells were counted in two random high power fields and this was expressed as a mean of the two counts. The S100-stained sections were scored by counting the number of positively stained dendritic cells within two random high power fields of the tumour and this was expressed as a mean: none present = 0, Less than 2 = 1, Greater than 2 = 2.

### Results

Results of staining with the antibodies are shown in Figures 1–3. The majority of tumours showed strong staining with AUA1 (cluster 2). There was no difference between the LTS and STS groups. LP34 was generally negative, showing no discrimination in the two groups. HMFG2 showed equal and variable expression in tumours from both LTS and STS groups with 54% of cases showing no expression at all. MBR1 showed a pattern of staining and overall results very similar to those of HMFG2. CEA was expressed in less than 50% of tumours in the study and there was no difference in staining pattern between LTS and STS groups.

NSE was demonstrated in less than 50% of cases overall and those which did stain showed similar results for both the LTS and the STS group (Figure 2). Chromagranin expression was present in approximately 40% of cases and the distribution of positive cases showed no difference between the LTS and STS groups (Figure 2). Our previous report with HNK1 suggested that lack of staining may be associated with a poor prognosis (Sheppeard *et al.*, 1987), but the present results show no significant association of lack of staining with either the LTS or the STS group (Figure 2).

SWA20 expression on SCLC was variable (Figure 3) with no relationship to prognostic category. In a small number of cases in both study groups occasional S100 positive dendritic cells were seen (Table III). Results with PC10, an antibody to PCNA (proliferating cell nuclear antigen), were uninterpretable in most of our cases because of the nuclear crush artefact commonly present in fibre-optic biopsies of SCLC.

### Discussion

There have been many attempts to relate biological characteristics of SCLC to prognosis. Cytomorphological characteristics of the tumour have been examined (Burdon *et al.*, 1979; Carney *et al.*, 1981; Vollmer *et al.*, 1985; Hirsch *et al.*, 1988) but the evidence that the tumours with a large cell component have a worse prognosis is not convincing. Serum markers such as NSE (Harding *et al.*, 1990) are related to prognosis but appear to be indicators of mass of disease rather than independent predictors of outcome. The same has been suggested for CEA (Sculier *et al.*, 1985; Laberge *et al.*, 1987), although a recent analysis of surgically resected tumours suggests that presence of CEA is an adverse prognostic feature independent of stage (Ruckdeschel *et al.* 1991). Other aspects of immunophenotype have been studied by Allan *et al.* (1987) who provided some evidence that expression of HMFG2 might be associated with a worse prognosis, and Hamid *et al.* (1987) who claimed a five fold increase in survival in surgically resected SCLC which did not show staining with an intibody to the C terminal peptide of human probombesin, also Martignone *et al.* (1989) who demon-

**Table II** Antibodies used in initial screen, and those (*) used in definitive assessment

| *Antibody* | *Type* | *Source* | *Antigen* |
|---|---|---|---|
| MOC21 | mAb | de Leij | Cluster 1a |
| 123C3 | mAb | Mooi | Probably Cluster 1a |
| AUA1* | mAb | ICRF | Cluster 2 |
| HMFG2* | mAb | ICRF | MUC Glycoprotein |
| SM3 | mAb | ICRF | Stripped protein core of MUC |
| HNK1* | mAb | ICRF | NCAM but not Cluster 1 |
| LP34* | mAb | ICRF | Cytokeratin |
| PC10* | mAb | ICRF | Proliferating cell nuclear antigen |
| HNK901 | mAb | Griffin | NCAM but not Cluster 1 |
| SWA4 | mAb | Stahel | Cluster 5 |
| SWA20* | mAb | Stahel | Clsuter 5a |
| B5 | mAb | Freedman | Proliferation marker on B cells |
| MBR1* | mAb | Menard | Membrane glycolipid on breast cancer and normal breast epithelial cells |
| CEA* | mAb | Dako | CEA |
| S100* | poly | Dako | S100 protein |
| NSE* | poly | Dako | Neurone specific enolase |
| Chromagranin* | mAb | Dako | Neuroscretory granule protein |

*Abbreviations*: MUC: Mucin. NCAM: Neural cell adhesion molecule. CEA: Carcino-embryonic antigen. NSE: Neurone-specific enolase. Cluster 1 (etc) refers to notations of the two small cell lung cancer antigen workshops (Souhami *et al.*, 1991).

**Figure 1** Each set of histograms shows the % of cases which fell into each of the 4 categories of % cells stained. Solid bars are long term survivors (LTS) and hatched bars are short term survivors (STS). Data are for AUA1, LF34, HMFG2, MBRI, CEA. The momenclature of the antibodies is given in Table II.

**Figure 2** Each set of histograms shows the % of cases which fell into each of the four categories of % cells stained. Solid bars are long term survivors (LTS) and hatched bars are short term survivors (STS). Data are for NSE, Chromogranin (CHR) and HNK1.

strated an inverse association between expression of the antigen recognised by MRB1 and overall survival in 63 unselected cases of SCLC.

There are many hidden biases in retrospective analyses of prognosis in cancer. In the usual analysis, tumours from a heterogenous group of patients, who have been treated in many different ways, are examined and the results compared with outcome. Such analyses often are unable to exclude treatment effects or the effects of case selection, and frequently do not indicate if the measurement has been made without knowledge of outcome or how missing data have been handled. Multiple analyses on the same data set add to the likelihood of chance, and false, associations.

In the present study we have tried to avoid some of these difficulties. The groups of patients have been taken at the extremes of prognosis (survival < 3 months or > 2 years) since, if no difference is detectable, it seems unlikely that one will be detected from a population with a wide distribution

**Figure 3** Each set of histograms shows the % of cases which fell into each of the four categories of % cells stained. Solid bars are long term survivors (LTS) and hatched bars are short term survivors (STS). Data are for antibody SWA20.

**Table III** Number of S100 positive dendrite cells within tumours as either negative, less than two per high power field, or more than two high power field. The first column shows the results in long term survivors (LTS) and the second column in short term survivors (STS)

| | *S100* | |
|---|---|---|
| | *LTS* | *STS* |
| Neg | 25 | 37 |
| <2/HPF | 8 | 2 |
| >2/HPF | 3 | 2 |

of prognosis. Each short survivor was the next age and sex matched case entered into the same treatment trial as the long survivor. In this way we have attempted to minimise treatment effects. The patients whose specimens were adequate appeared to be representative of the larger group as judged by age and sex. Finally, the slides were read and scored by three independent observers without knowledge of outcome.

Antibodies used in this study were selected to include markers previously reported as being related to prognosis in SCLC and reagents recognising neuroendocrine and epithelial antigens previously identified on SCLC. AUA1 binds to a 40Kd membrane glycoprotein (Spurr *et al.*, 1986; Strnad *et al.*, 1989), designated as Cluster 2 in the Lung Cancer Antibody Workshops (Souhami *et al.*, 1991). The antigen appears to be a homologue of the cell adhesion protein nidogen (Simon *et al.*, 1990). LP34 recognises cytokeratins 5, 8, 16, 10 and 14, which are expressed by epithelia showing squamous and glandular differentiation (Lane & Alexander, 1990).

HMFG2 recognises an epitope on a large 180Kd epithelial mucin which is expressed on normal and lactating breast epithelium and breast carcinoma cells. The lack of expression of this antigen in over fifty per cent of our cases is of interest since it has been a target antigen for immunolocalisation (Epenetos *et al.*, 1982). MBR1, like HMFG2, was initially raised and studied in the context of breast carcinoma.

CEA is one of the most widely studied tumour-associated antigens which has recently been shown to belong to the immunoglobulin superfamily of cell adhesion molecules (Pignatelli *et al.*, 1990). It is strongly expressed in a wide range of adenocarcinomas (Sheahan *et al.*, 1990), and in just over half of small cell lung cancers (Bepler *et al.*, 1989). The gamma dimer of NSE, a glycolytic enzyme found in the brain, has been widely used as a marker of neuroendocrine differentiation in tumours (Sheppard *et al.*, 1984). Chromogranin A is a soluble protein in dense core granules and has been demonstrated immunohistochemically in approxim ately 50% of SCLC in most reported series (Wilson & Lloyd, 1984). HNK1 has been shown to be expressed on SCLC, carcinoids, some adenocarcinomas, natural killer cells and neural tissues, and belongs to the CD54 category of leucocyte differentiation antigens which is an epitope of NCAM (Kruse *et al.*, 1984).

SWA20 (designated as Cluster 5) is an antibody which shows a mixed neural and epithelial reactivity (Waibel *et al.*, 1988). S100 is a brain protein which has a wide tissue distribution including Langerhans cells. Several studies on the presence and extent of these cells within other malignant tumours, and the relationship of their distribution to prognosis, have been carried out (Fox *et al.*, 1989).

The results of this study do not confirm previous findings (Allan *et al.*, 1987; Hamid *et al.*, 1987; Martignone *et al.*, 1989, Ruckdeschel *et al.*, 1991) that the use of immunohistochemistry can identify patients with a good or bad prognosis with SCLC. This negative result must itself be interpreted cautiously. Firstly we examined mainly fibre-optic bronchoscopy specimens which, of course, may not represent the whole tumour. Nevertheless these are the specimens which the pathologist receives in the vast majority of cases and on which the clinician makes a judgement about treatment. Prognostic indicators based on surgically resected SCLC will be of very limited value. Secondly the panel clearly does not represent all possible antigens on SCLC. The neural cell adhesion molecule (NCAM) designated as Cluster 1 in the Lung Cancer Workshops (Souhami *et al.*, 1991) is a marker indicative of the neuroendocrine status of the tumour. Unfortunately it is lost in formalin fixation unless zinc sulphate is added (Tome *et al.*, 1990) and so could not be used in this study. Nevertheless Chromogranin, HNK1 and NSE are neuroendocrine markers and showed no relationship to prognosis. This data from the present study cannot however be regarded as conclusive on this point and further, prospective, studies are needed.

For the present it does not appear that any immunohistochemical staining can be recommended as a valuable addition to the routine diagnosis of SCLC, or to the other clinical and investigational findings on which a judgement about prognosis and treatment is made.

This work was supported in part by the United Kingdom Coordinating Committee on Cancer Research. The authors would like to thank Dr S. Menard, Dr R. Stahel and Dr D. Lane for supplying the antibodies MBR1, SWA4, SWA20 and PC10 respectively, and Mrs Maureen Cohen for typing the manuscript.

### References

AISNER, J., ALBERTO, P., BITRAN, J. & 5 others (1983). Role of chemotherapy in small cell lung cancer. *Cancer Treat. Rep.*, **67**, 37.

ALLAN, S.G., HAY, F.G., MCINTYRE, M.A. & LEONARD, R.C. (1987). Prognosis of small cell carcinoma of the lung -relationship to human milk fat globule 2 [HMFG2] antigen and other small cell associated antigens. *Br. J. Cancer*, **56**, 485–488.

BEPLER, G., OSTHOLT, M., NEUMANN, K. & 4 others (1989). Carcinoembryonic antigen as differentiation marker for small cell lung cancer *in vitro* and its clinical relevance. *Anticancer Res.*, **9**, 1525.

BURDON, J.G.W., SINCLAIR, R.A. & HENDERSON, M.M. (1979). Small cell carcinoma of the lung: Prognosis in relation to histologic subtypes. *Chest*, **76**, 302–304.

CARNEY, D.N., MATTHEWS, M.J., IHDE, D.C. & 5 others (1981). Influence of histological subtype of small cell carcinoma of the lung on clinical presentation, response to therapy and survival. *J. Natl Cancer. Inst.*, **65**, 1225.

DAVIS, S., WRIGHT, P.W., SCHULMAN, S.F., SCHOLES, D., THORNING, D. & HAMMAR, S. (1985). Long term survival in small cell carcinoma of the lung: a popular experience. *J. Clin. Oncol.*, **3**, 80.

EPENETOS, A.A., BRITTON, K.E., MATHER, S. & 8 others (1982). Targetting of iodine-123-labelled tumour-associated monoclonal antibodies to ovarian, breast and gastro-intestinal tumours. *Lancet*, **ii**, 999.

FOX, S.B., JONES, M., DUNNILL, M.S., GATTER, K.C. & MASON, D.Y. (1989). Langerhans cells in human lung tumours: an immunohistological study. *Histopathology*, **14**, 269–275.

HALL, P.A., LEVISON, D.A., WOODS, A.L. & 9 others (1990). Proliferating cell nuclear antigen (PCNA) immunolocalization in paraffin sections: an index of cell proliferation with evidence of deregulated expression in some neoplasms. *J. Path.*, **162**, 285.

HAMID, Q.A., ADDIS, B.J., SPRINGALL, D.R. & 4 others (1987). Expression of the C-terminal peptide of human pro-bombesin in 361 lung endocrine tumours, a reliable marker and possible prognostic indicator for small cell carcinoma. *Virchows Arch. A.*, **411**, 185.

HANSEN, H.H., DOMBERNOWSKY, P. & HIRSCH, F.R. (1978). Staging procedures and prognostic features in small cell anaplastic bronchogenic carcinoma. *Semin. Oncol.*, **5**, 280–287.

HARDING, M., MCALLISTER, J., HULKS, G. & 4 others (1990). Neurone specific enolase (NSE) in small cell lung cancer: a tumour marker of prognostic significance? *Br. J. Cancer*, **61**, 605.

HIRSCH, F.R., MATTHEWS, M.D., AISNER, S. & 9 others (1988) Histopathologic classification of small cell lung cancer. Changing concepts and terminology. *Cancer*, **62**, 973.

KRUSE, J., MAILHAMMER, R., WENNECKE, H. & 4 others (1984). Neural cell adhesion molecules and MAG share a common carbohydrate moeity recognised by monoclonal antibodies L2 and HNK1. *Nature*, **311**, 153.

LABERGE, F., FRITSCHE, H.A., UMSAWASDI, T. & 9 others (1987). Use of carcinoembryonic antigen in small cell lung cancer. Prognostic value and relation to the clinical course. *Cancer*, **59**, 2047.

LANE, E.B. & ALEXANDER, C.M. (1990). Use of keratin antibodies in tumor diagnosis. *Cancer Biol.*, **1**, 165–179.

MARTIGNONE, S., BEDINI, A.V., CIAVOLELLA, A. & 6 others (1989). Relationship between CaMBr1 expression and tumor progression in small cell lung carcinomas. *Tumori*, **75**, 373.

PIGNATELLI, M., DURBIN, H. & BODMER, W.F. (1990). Carcinoembryonic antigen functions as an accessory adhesion molecule mediating colon epithelial cell-collagen interactions. *Proc. Natl Acad. Sci. USA*, **87**, 1541–1545.

RAWSON, N.S.B. & PETO, J. (1990). An overview of prognostic factors in small cell lung cancer. *Br. J. Cancer*, **61**, 597–604.

SCULIER, J.P., FELD, R., EVANS, W.K. & 4 others (1985). Carcinoembryonic antigen: A useful prognostic marker in small cell lung cancer. *J. Clin. Oncol.*, **3**, 1349.

SHEAHAN, K., O'BRIEN, M., BURKE, B. & 4 others (1990). Differential reactivities of carcinoembryonic antigen (CEA) and CEA-related monoclonal and polyclonal antibodies in common epithelial malignancies. *Am. J. Clin. Pathol.*, **94**, 157.

SHEPPARD, M.N., CORRIN, B., BENNETT, M.H., MARANGOS, P.J., BLOOM, S.R., & POLAK, J.M. (1984). Immunocytochemical localization of neuron specific enolase in small cell carcinomas and carcinoid tumours of the lung. *Histopathology*, **8**, 171–181.

SHEPPARD, M.N., MORITTU, L., ADDIS, B., SOUHAMI, R.L. & BOBROW, L.G. (1987). Analysis of antigenic phenotype in small cell lung carcinoma in patients with long and short term survival. *J. Pathol.*, **151**, 59A.

SIMON, B., PODOLSKY, D.K., MOLDENHAUER & 3 others (1990). Epithelial glycoprotein is a member of a family of epithelial cell surface antigens homologous to nidogen, a matrix adhesion protein. *Proc. Natl Acad. Scie. USA*, **87**, 2755.

SKOV, B.G., SORENSEN, J.B., HIRSCH, F.R., LARSSON, L.I. & HANSEN, H.H. (1991). Prognostic impact of histologic demonstration of chromagranin A and neuron specific enolase in pulmonary adenocarcinoma. *Ann. Oncol.*, **2**, 355–360.

SOOMRO, I.N. & WHIMSTER, W.F. (1990). Growth fraction in lung tumours determined by Ki67 immunostaining and comparison with AgNOR scores. *J. Path.*, **162**, 217–222.

SPURR, N.K., DURBIN, H., SHEER, D., PARKAR, M., BOBROW, L. & BODMER, W.F. (1986). Characterisation and chromosomal assignment of a human cell surface antigen defined by RHE monoclonal antibody AUA1. *Int. J. Cancer*, **38**, 631–636.

SOUHAMI, R.L., BEVERLEY, P.C.L. & BOBROW, L.G. (1987). Antigens of small cell lung cancer. First International Workshop. *Lancet*, **ii**, 325–326.

SOUHAMI, R.L., BEVERLEY, P.C.L., BOBROW, L.G. & LEDERMANN, J.A. (1991). Results of central data analysis: 2nd International Workshop on Small Cell Lung Cancer Antigens. *J. Natl Cancer Inst.*, **83**, 609–612.

SOUHAMI, R.L., BRADBURY, J., GEDDES, D.M., SPIRO, S.G., HARPER, P.G. & TOBIAS, J.S. (1985). Prognostic significance of laboratory parameters measured at diagnosis in small cell carcinoma of the lung. *Cancer Res.*, **45**, 2878–2882.

SOUHAMI, R.L. & LAW, K. (1990). Longevity in small cell lung cancer. *Brit. J. Cancer*, **61**, 584–589.

STRNAD, J., HAMILTON, A.E., BEAVERS, L.S. & 7 others (1989). Molecular cloning and characterisation of a human adenocarconoma/epithelial cell surface antigen complementary DNA. *Cancer Res.*, **49**, 314.

TOME, Y., HIROHASHI, S., NOGUCHI, M. & SHIMOSATO, Y. (1990). Preservation of cluster 1 small cell lung cancer antigen in zinc-formalin fixative and its application to immunohistological diagnosis. *Histopathology*, **16**, 469–470.

VOLLMER, R.T., BIRCH, R., OGDEN, L. & CRISSMAN, J.D. (1985). Subclassification of small cell cancer of the lung. The Southeastern Cancer Study Group experience. *Human Pathol.*, **16**, 247–252.

WAIBEL, R., O'HARA, C.J., SMITH, A. & STAHEL, R.A. (1988). Tumor associated membrane sialoglycoprotein on human small cell lung carcinoma identified by the IgG2a monoclonal antibody SWA20. *Cancer Res.*, **48**, 4318–4323.

WILSON, B.S. & LLOYD, R.V. (1984). Detection of chromogranin in neuroendocrine cells with a monoclonal antibody. *Am. J. Pathol.*, **115**, 458–468.